# New Drugs, Appliances, and Things Medical

**Published:** 1891-08-08

**Authors:** 


### NEW DRUGS, APPLIANCES, AND THINGS MEDICAL.

[All preparations, appliances, novelties, etc., of which a notice is desired, should be sent for The Editor, to care of The Manager, 140, Strand, London, W.C.]

### KOPS' ALE.

Wandsworth Bridge Road, Fulham.

We have examined samples of this beverage. It is stated to be non-alcoholic, and to be brewed from the best hops. We find that these statements are correct. It is well aërated, tastes clean, and is the nearest approach to ordinary alcoholic ale that we have met with. Those requiring a bright effervescing drink, with a bitter stomachic quality, will find Kop's ale an excellent drink. The hop flavour is particularly well marked, in fact, it leaves the impression on the tongue of freshly-dried hops.

### PATENT STOPPER FOR POISONS.

From Messrs. Calvert, Bradford, Manchester, the well-known manufacturers of carbolic acid, we have received a sample of the above. The firm has adopted it in order to lessen the chance of accidental poisoning by carbolic preparations. The stopper is manufactured by the Patent Stopper and Stamp Company, Birmingham. It consists of a metallic, many-pointed star, which is fixed to the top of the stopper or cork of the bottle. The use of this makes it impossible to remove the cork without recognizing the fact that it is that of a poison bottle. If the cork or stopper is firmly fixed it is impossible to remove it without placing a cloth round it, as the star angles are so sharp as to be very unpleasant to the hand. We think Messrs. Calvert have succeeded admirably in placing such a "pointed" warning at the mouths of their bottles. It should be noted that it will serve equally well for other poisonous substances which are kept in bottles.

### ALFRED CARTER'S INVALID FURNITURE AND APPLIANCES.

47, Holborn Viaduct.

Mr. Alfred Carter has again forwarded his catalogue to us with its long and useful list of appliances for the sick, the halt, the maimed, and the blind. We gave last year a long account of a visit we paid to his warehouse on Holborn Viaduct, and of his many admirable inventions there on view. This catalogue presents in a compact form all the information required for fitting up any sick-room, or for obtaining chairs, crutches, or mechanical aids of any sort. It is well illustrated, so that one can see at a glance the nature of the apparatus one is about to obtain. We note that Mr. Carter was honoured by the order for the Bath chair used by Her Majesty at the various late exhibitions. When a man supplies the Court, both Services, the Courts of Justice, the Colonial (Crown) Agents, Board of works, and is the inventor of the ambulance appliances which are used by the Hospitals Association all over the metropolis, his character for good work is too well established to need further praise. We can only say, as we did before, that we know of no establishment where a completer invalid outfit can be obtained.

### SODA-WATER.

Between the years 1770 and 1783 Joseph Priestly made many investigations into the properties of gases. Oxygen was discovered by him in 1772, but though this was a remarkable point in the history of the chemistry of gases, yet he has conferred greater benefits on the human race by his studies in the properties of carbonic acid. It happened that he lived in the neighbourhood of a brewery. During the process of brewing large quantities of carbonic acid gas are given off. Only recently a company has been started to utilize this waste gas. It is calculated that thus it can be compressed and sold at an enormously cheaper rate than if it be produced from chemicals. This, we expect, will in turn make aërated waters even cheaper than they are. Priestly found that this "fixed air," as he termed it, was soluble in water. If he left a shallow dish of water over night in the layer of gas above the fermenting liquor, it was next morning sparkling and gently acidulous in taste, like the then fashionable mineral water called Pyrmont. This simple experiment was the germ of soda-water manufacture. A Dr. Nooth about this time invented the first gazogene, which was improved by a Mr. Parker. For many years this was the only way in which soda-water could be obtained for domestic use. We may here mention that carbonic acid was tried in many diseases as a remedial agent. In Priestly's works are numerous cases narrated by some of the foremost practitioners of the day. It is described as particularly effective in stilling pain and in making offensive inflammatory discharges sweet. After Nooth's machine followed the many varieties of domestic soda-water generators, such as we commonly see in private houses, and which go by the names of gazogene and seltzogene. Soda-water in bottles came in during the first part of the century. The caricaturists of the time did not omit to bring the novel drink into their skits. We all remember the dismay of Handy Andy, who discharged a bottle of soda on the company assembled at dinner, and then exclaimed, "Holy Moses! I niver saw boiling cold water before." For years one saw nothing but the little bottles "what would not stand upright" with tightly tied down corks. Then wire superseded string, followed by the varieties of ball valves, and last of all by the patent screw stopper. We have thus hurriedly sketched the history of soda-water as preliminary to the latest development of its manufacture. This is to be seen at Chinnery and Co.'s offices, 1, Leadenhall Street, E.C. We this year gave a detailed account of their works, and may briefly recapitulate the advantages of using their machines.

1. Cheapness. With chemicals a dozen bottles can be produced for less than twopence. With compressed carbonic acid gas the cost is from a halfpenny to a penny.
2. Purity. The machines are all lined with glass or enamel. Therefore there is no chance of metallic contamination.
3. Strength. The machine is made of gun metal.
4. Adaptability. They can be either used directly as a domestic gazogene or for filling bottles or syphons.

Though, of course, the price is large as compared with that of a gazogene, yet where much aërated water is used they will be found invaluable, especially in large houses, shooting lodges, and the like. Again, for chemists doing a retail trade the machine will be found the cheapest in the market for filling syphons. The same holds good for hotels, confectioners, and restaurants.

We fancy that colonists will find a Chinnery's machine a great boon, as with chemicals a large supply of aërated water can be had for a very slight cost, doing away with the necessity of the carriage of the bulky and heavy bottle boxes. Given a machine, and a gross or so of bottles with the chemicals, and any out-of-the-way station or house is permanently set up with the luxury of aërated waters.

We may add that the machine can aërate water with any gas that may be desired to be used. Thus oxygen can be administered in the pleasant form of oxygenated water, which is much given for diabetes and so on.

